# Effects of Extraction Methods on Physicochemical and Structural Properties of Common Vetch Starch

**DOI:** 10.3390/foods11182920

**Published:** 2022-09-19

**Authors:** Xiaojun Zhang, Yongqiang Cheng, Xin Jia, Donghui Geng, Xiaojia Bian, Ning Tang

**Affiliations:** 1 College of Food Science and Nutritional Engineering, China Agricultural University, Beijing 100083, China; 2 Beijing Key Laboratory of Functional Food from Plant Resources, Beijing 100083, China

**Keywords:** common vetch starch, back-slopping fermentation, physicochemical properties, structure

## Abstract

Three extraction methods: water extraction, lactic acid bacteria fermentation, and back-slopping fermentation were applied to extract a new type of legume starch, common vetch starch. Our results showed that the lactic acid bacteria fermented starch had the highest amylose content (35.69%), followed by the back-slopping fermented starch (32.34%), and the water-extracted starch (30.25%). Furthermore, erosion surface, lower molecular weight, smaller particle size, larger specific surface area, and a higher proportion of B1 chain were observed in the fermented starch, especially in the back-slopping fermented starch. All the extracted starches showed a type C structure, but a type C_B_ structure was observed in the back-slopping fermented starch. In addition, the relative crystallinity of the lactic acid bacteria fermented starch (34.16%) and the back-slopping fermented starch (39.43%) was significantly higher than that of the water-extracted starch (30.22%). Moreover, the swelling power, solubility, pasting, and thermal properties of the fermented starches were also improved. In conclusion, the fermentation extraction method, especially back-slopping fermentation, could improve the quality of the extracted common vetch starch when compared with the traditional water extraction method.

## 1. Introduction

Cereal, tuber, and legume starches are the main types of starches, and they are important sources of energy for humans [1]. Compared with the other common starches such as corn starch and potato starch, legume starch has fewer resources, but it is still widely used in the food industry due to its unique structural and functional properties [2]. Some studies have shown that these unique properties can be obtained through chemical modifications of potato and tapioca starches. The required chemicals and consumed energy for this process, however, are rarely green [3,4]. Furthermore, some studies have also found that the combination of other materials and starch can improve the processing characteristics of starch [5], but the high cost and poor stability limited the use of this method. The development of new starches has always been considered to be a good strategy for this problem [6]; therefore, it is very necessary to expand the resources of legume starch. Common vetch (*Vicia sativa* L.) is an annual high-quality legume crop widely grown worldwide, especially in China, Canada, and Mediterranean countries [7,8,9]. It has received increasing attention from researchers due to its beneficial effect on the soil and contribution to maintaining ecosystems [10,11]. Moreover, the common vetch was found to have high levels of starch with a high gelatinization temperature and relatively low setback. Therefore, common vetch has great potential for the extraction of starch that could be used in the food industry.

To date, water extraction is the most widely used method for extracting starch from leguminous plants [12,13]. In recent years, lactic acid bacteria fermentation and back-slopping fermentation were also used for extracting starch as the fermentation method was considered to be a practicable tool for modifying starches [14,15]. Since the starches extracted by different methods have different structures and properties, the method used for starch extraction depends on the raw materials and the properties required for the starch application [15,16]. In general, the legume starches extracted by fermentation methods were more suitable for starch processing; however, the reasons are not clear [15,17,18]. In addition, previous studies have shown that the effects of fermentation on different starch resources were quite different [15,19]. Systematic studies on the effects of different extraction methods on the structure and properties of different types of starches are highly needed.

Accordingly, in the present study, three extraction methods: water extraction, lactic acid bacteria fermentation, and back-slopping fermentation were applied to extract the starch from common vetch (*Longjian No. 1*), which is widely grown in the Gansu province of China. The chemical composition, thermal and pasting properties, and morphological characteristics of the obtained starches were systematically evaluated to investigate the effects of the different extraction methods on common vetch starch.

## 2. Materials and Methods

### 2.1. Materials

The seeds of common vetch (*Longjian No. 1*) with uniform size and fully ripe were provided by the Gansu Planting Base (Wuwei, Gansu, China). The seed has the composition of 36.73 ± 2.33% protein, 45.58 ± 2.58% starch, 11.13 ± 0.66% moisture, 1.78 ± 0.05% fat, 4.27 ± 0.37% fiber, and 3.77 ± 0.02% ash. Dimethyl sulfoxide (DMSO) was chromatographically pure and purchased from Merck KGaA (Darmstadt, Germany). All other reagents and chemicals used in the study were reagent grade.

### 2.2. Starch Extraction

Common vetch seeds were washed and then mixed with distilled water at a ratio of 1:4, and left at room temperature for 4 h. Then the soaked seeds were ground using a homogenizer (L18-P132, Joyoung, Jinan, Shandong, China) to prepare the common vetch seed slurry, and the slurry was collected after being sieved through a 150-mesh sieve. For water extraction (WS), the common vetch starch was extracted according to the method of Li et al. [20]. Briefly, the obtained slurry was centrifuged at 5000× *g* for 25 min, and the supernatant was discarded. Then, the precipitate was washed 4 times using distilled water and the wet starch was obtained after being sieved with a 200-mesh sieve. The final starch sample was obtained by drying the wet starch in a hot air stream at 45 °C for 6 h. For the back-slopping fermentation method (NS), the starch was obtained according to the method of Fayemi et al. with some modifications [20]. Briefly, the slurry was mixed with the water that has been previously used for soaking the common vetch seeds (48 h) at a ratio of 20:1 (*v*/*v*), and then the mixture was fermented for 8 h at 37 °C and stirred every 1 h. After the fermentation, the starch extraction followed the water extraction procedure. For the lactic acid bacteria fermentation method (LS), the lactic acid bacteria was first isolated from the water previously used for soaking (48 h), and then the slurry was mixed with the isolated lactic acid bacteria (8.78 log_10_ CFU/mL) at a ratio of 20:1, fermented at 37 °C for 8 h, and stirred every 1 h. After the fermentation, the starch extraction followed the water extraction procedure. 

### 2.3. Chemical Composition

Moisture, lipid, and protein content of the starches extracted by the different methods were determined according to the methods of AACCI and AOAC [21]. The total starch and amylose content was determined using total starch and amylose/amylopectin assay kits (Megazyme, Wicklow, Ireland) [21].

### 2.4. Morphological Characteristics

The starch powder was evenly sprinkled on a double-sided carbon tape and coated with gold-palladium. Morphological pictures of the starch granules were obtained using a scanning electron microscope (GeminiSEM 500, Carl Zeiss, Jena, Thüringen, Germany) with an accelerating voltage of 10.0 kV and a magnification of 500× and 1000×. 

### 2.5. Particle Size Distribution

The starch samples were uniformly dispersed in distilled water, and the particle size distribution of starch samples was measured using a Malvern Mastersizer 2000 (Malvern, Malvern Panalytical, UK) with the refractive index set to 1.52 and absorbance set to 0.1.

### 2.6. Molecular Weight and Amylopectin Chain-Length Distributions

The homogeneity and molecular weight of the starch samples were measured using SEC-MALLS-RI [22]. The weight- and number-average molecular weight (Mw and Mn) and polydispersity index (Mw/Mn) of the starch samples in DMSO/LiBr (0.5% *w*/*w*) solution were measured on a DAWN HELEOS-II laser photometer (He-Ne laser, λ = 663.7 nm, Wyatt Technology Co., Goleta, CA, USA) equipped with three tandem columns (300 × 8 mm, Shodex OH-Pak SB-805, 804 and 803; Showa Denko K.K., Tokyo, Japan), which was held at 60 °C using a model column heater. The flow rate was 0.3 mL/min. Data were acquired and processed using ASTRA6.1 (ASTRA 6.1, Wyatt Technology Corporation, Santa Barbara, CA, USA). The amylopectin chain-length distributions were analyzed by high-performance anion-exchange chromatography on a CarboPac PA 100 anion-exchange column with a pulsed amperometric detector (ICS 5000, Thermo Fisher Inc., Waltham, MA, USA) [23]. Starch (5 mg) was mixed with 5 mL of water and then boiled for 60 min; the obtained starch dispersion was mixed with sodium azide solution (10 μL, 2% *w*/*v*), acetate buffer (50 μL, 0.6 M, pH 4.4), and isoamylase (10 μL, 1400 U) and incubated at 37 °C for 24 h. The above mixture was then mixed with sodium borohydride 0.5% (*w*/*v*) and left at room temperature for 20 h; 600 μL of the above mixture was vacuum dried at room temperature and then dissolved in 20 μL of 1 M NaOH. Finally, the mixture was diluted with 580 μL of distilled water before injection. The parameter settings of the HPAEC-PAD were adopted according to the previously established method [24]. 

### 2.7. X-ray Diffraction

The crystalline structure of the starch was analyzed using a D8 Advance X-ray diffractometer (XRD) (BRUKER AXS GMBH, Karlsruhe, Germany) with Ni-filtered Cu Ka radiation (35 kV, 20 mA). The X-ray diffractogram was recorded by scanning from the diffraction angle (2θ) of 4°–45° at a scanning speed of 3°/min and a step size of 0.02°. The relative crystallinity of starch was calculated using JADE software 5.0 (Materials Date Inc., Santa Ana, CA, USA) [25].

### 2.8. Fourier Transform Infrared Spectroscopy (FT-IR) and Raman Spectroscopy

The FT-IR spectra of starch samples were measured using a Fourier transform infrared spectrometer (Vertex 70, Bruker, Bremen, Germany). The starch samples were prepared at the ratio of 1 g starch/100 g KBr and then compressed into pellets. The spectra were recorded between 4000 cm^−1^ and 400 cm^−1^ with a scan speed of 4 cm^−1^. The Raman spectra of starch samples were measured using a Renishaw inVia confocal Raman microscope (Renishaw, Wotton-under-Edge, UK) with a 785 nm green diode laser source [26]. The spectra were recorded between 3200 cm^−1^ and 100 cm^−1^ with a scan speed of 7 cm^−1^.

### 2.9. ^13^C CP/MAS NMR Analysis

The ^13^C CP/MAS NMR analysis of the starch was performed on a JNM-ECZ600R spectrometer (JEOL Ltd., Tokyo, Japan); the frequency of 150.913 MHz, tube diameter of 3.2 mm, magic-angle spinning frequency of 12 kHz, relaxation delay of 2 s, and scans of 1221 were used during the experiments. The specific relative crystallinity, double helix content, and the amorphous phase were calculated according to previously established methods [27,28]. 

### 2.10. Pasting Property

The pasting properties of starch were evaluated using a rapid viscometer (RVA 4500, Perten, Stockholm, Sweden) [29]. The 10% (*w*/*w*) starch slurry was used for the detection; the initial temperature was set to 50 °C, then heated to 95 °C at a rate of 7.5 °C/min for 5 min, and then cooled to 50 °C at a rate of 7.5 °C/min for 1 min.

### 2.11. Thermal Property 

The thermal property of the starch was detected by a differential scanning calorimeter (DSC-Q-2000, TA Instruments, New Castle, DE, USA) according to the method of Wang et al., with slight modifications [30]. Briefly, 2.5 μg of starch was mixed with 7.5 μL of deionized water and sealed in an aluminum crucible, and equilibrated at room temperature for 24 h. After the equilibrium, the starting temperature of 30 °C, the nitrogen flow rate of 20–50 mL/min, and the temperature raised to 100 °C at a rate of 10 °C/min were used for determination. 

### 2.12. Starch Solubility and Swelling Power

The starch solution (2%, *w*/*v*) was continuously stirred in a 90 °C water bath for 30 min, then cooled to room temperature and centrifuged at 4000 rpm for 20 min. The supernatant was poured out in an aluminum box and dried at 105 °C for 3 h. Then, the weight of the precipitate after centrifugation (m_2_) and the starch in the aluminum box (m_1_) were weighed, respectively. Then, the solubility (SA) and swelling power (SP) were calculated according to the following formulas:

SA (%) = m_1_/m_2_ × 100% (1)

SP (%) = m_2_/(m_2_ − m_1_) × 100% (2)

### 2.13. Statistical Analysis

All measurements were performed in triplicate. The statistics were calculated using SPSS (version 22, IBM, Armonk, NY, USA) and Origin software (version 9.1, OriginLab, Northampton, MA, USA). The data were expressed as mean ± standard deviation and the difference was considered to be at the 95% level of significance (*p* < 0.05) using one-way ANOVA with Tukey adjustment. Pearson correlations were also analyzed using SPSS (version 22, IBM, Armonk, NY, USA) at the level of *p* < 0.05 and *p* < 0.01 for significant and quite significant correlations, respectively.

## 3. Results and Discussion

### 3.1. Chemical Compositions

The composition of the starches extracted by water extraction, lactic acid bacteria fermentation, and back-slopping fermentation is shown in Table 1. The total starch contents of the obtained starches were similar and all above 90%. Furthermore, the moisture and protein contents between starches were also similar, but the lipid contents of the starches extracted by water extraction and lactic acid bacteria fermentation were significantly higher than the starch extracted by back-slopping fermentation. This may be attributed to more enzymes that are related to fat metabolism can be produced during natural fermentation [31]. The same phenomenon was also observed in naturally fermented mung bean starch [32]. In addition, the proportion of amylose in the fermented starch was significantly increased compared with the starch extracted by water extraction, especially in the starch extracted by lactic acid bacteria fermentation due to the depolymerization of the short outer chains of amylopectin [19,33]. 

### 3.2. Morphology Structure and Particle Size Distribution of Starch Granules

**Figure 1:**
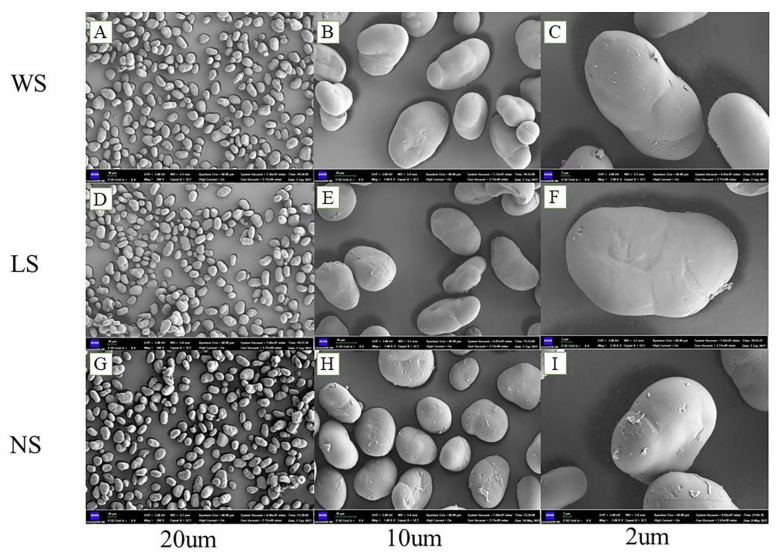
Scanning electron micrographs of starch samples extracted by different methods. (**A**–**C**) are images of WS starch magnified at 200×, 1000×, and 2000×, respectively. (**D**–**F**) are images of LS starch magnified at 200×, 1000×, and 2000×, respectively. (**G**–**I**) are images of NS starch magnified at 200×, 1000×, and 2000×, respectively.

The morphologies of the starch granules are shown in Figure 1, and all the starch granules exhibited a spherical or ellipsoidal shape with relatively uniform size, but there were obvious differences in the roughness of the starch surface. The roughness of the starch surface increased after fermentation, especially in the back-slopping fermented starch suggesting that more starch surfaces were destroyed during back-slopping fermentation. This was in agreement with previous studies [16]. Table 2 shows the particle size distribution of the obtained starches. As can be seen from Table 2, the particle size distribution of starches extracted by water extraction, lactic acid bacteria fermentation, and back-slopping fermentation ranged from 18.31 to 43.56 μm, 18.25 to 42.42 μm, and 12.74 to 38.60 μm, respectively. It was suggested that the fermentation method could produce smaller starch particle size, and the value of D(3,2) and specific surface area (SSA) indicated that the surface of starch granules extracted by fermentation was eroded, and more or larger micropores were formed for the starch granules extracted by back-slopping fermentation. In addition, the high damage of the starch structure caused by the back-slopping fermentation method would lead to a relatively smaller particle size. These results were consistent with the scanning electron microscopy results.

### 3.3. Molecular Weight and Chain Length Distribution of the Starches

**Figure 2:**
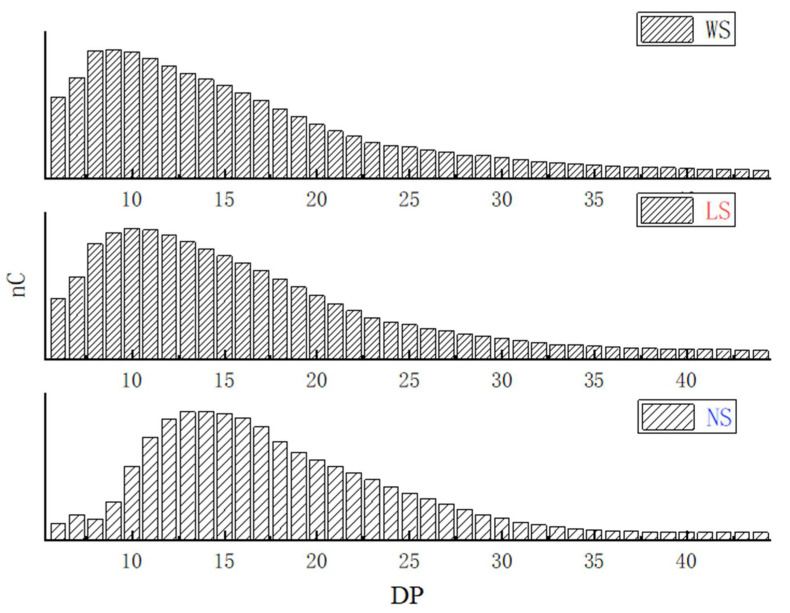
Amylopectin chain length distribution of starch samples extracted by different methods.

The weight-average molecular weight (M_w_) and number-average molecular weight (M_n_) of three starches are presented in Table 2. The M_w_ and M_n_ of the starches extracted by fermentation were significantly lower, especially the starch extracted by natural fermentation. These results indicated that the common vetch starch granules were degraded during the fermentation extraction, and the decomposition of starch by natural bacteria may be stronger than that of lactic acid bacteria. Similar results were also observed for fermented corn starch, potato starch, and tapioca starch [16,34]. According to previous studies, the chain length was divided into the following four parts: A chain (DP 6–12), B1 chain (DP 13–24), B2 chain (DP 25–36), and B3 chain (DP ≥ 37) [24]. As can be seen from Table 2 and Figure 2, the proportions of A chains in starches extracted by fermentation were significantly lower than that in the starch extracted by water extraction. The proportion of B2 and B3 chains in the starch extracted by lactic acid bacteria fermentation was slightly lower than that in the starch extracted by water extraction. This may be due to part to the long amylopectin in the amorphous region that was decomposed by amylase and organic acids produced by lactic acid bacteria fermentation [35,36]. However, the abundance of B2 and B3 in the back-slopping fermented starch was significantly higher than that in the starches extracted by the other methods, suggesting that back-slopping fermentation may affect more of the crystalline region of starch. Similar results were also observed in mung bean starches extracted by different methods [14].

### 3.4. Crystalline Structure

**Figure 3:**
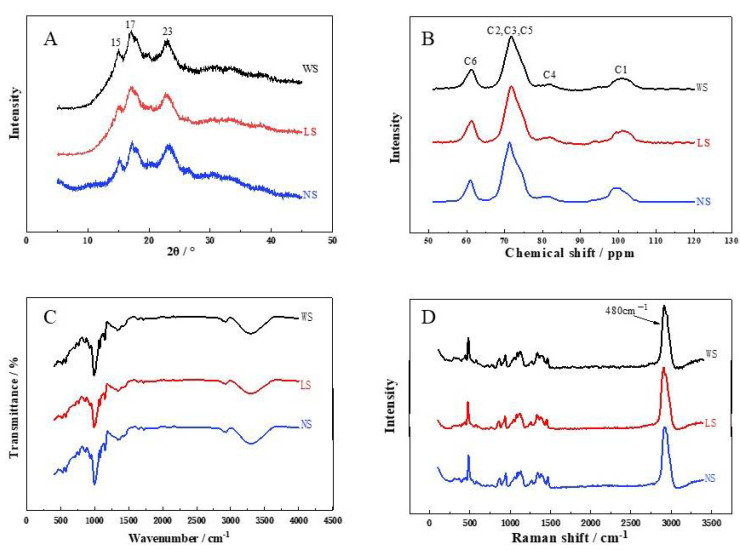
Crystal structure of starch samples extracted by different methods. (**A**) is the result of XRD diffraction patterns, (**B**) is the result of ^13^C CP/MPS NMR spectra, (**C**) is the result of the FT-IR deconvoluted spectra, and (**D**) is the result of the Raman spectrum.

As illustrated in Table 3 and Figure 3A, three obvious peaks at the 2θ values of 15°, 17°, and 23° were observed in all starches, indicating that all starches were typical crystals with type C. However, the relative crystallization of starches extracted by back-slopping fermentation (39.43), lactic acid fermentation (34.16), and water extraction (30.22) was significantly different, suggesting that the amorphous area of the starch was destroyed by the acid and amylase generated by fermentation [16,25]. In addition, the relative crystallization of the starch extracted by back-slopping fermentation was higher compared with the starch extracted by lactic acid bacteria fermentation, and this may be due to the differences in the proportion of the B1 chain in starches as the amylopectin with a polymerization degree of 14–24 or 12–22 is more likely to form a double helix structure [37].

The ^13^C cross-polarization/magic angle spinning NMR is an advantageous way to analyze the helical and crystal structure of starch, especially the short-range ordered structure of starch molecules. This technique was also applied to investigate the structures of the obtained starches, and the results are shown in Table 3 and Figure 3B. The NMR spectra of the three starches were similar and were divided into C_1_, C_2,3,5_, C_4_, and C_6_ regions [38]. After fermentation, the proportion of the amorphous region and the content of the single helical structure decreased, while the content of the double helical structure and crystallinity increased. Compared with the starch fermented by lactic acid bacteria, the content of double helix structure and relative crystallinity of the back-slopping fermented starch is higher, while the proportion of the amorphous region and the content of the single helix structure was lower. These results were in agreement with the XRD results, which also suggested that the amorphous region of the starch was destroyed after fermentation. However, the C1 spectrum of the starch extracted by back-slopping fermentation showed double peaks, and was different from that of the other starches, indicating that its C-type crystals were dominated by B-type crystals. Similar results were also observed in other fermented starches [37,39].

The ^13^C cross-polarization/magic angle spinning NMR, FT-IR and Raman spectroscopy are two well-established tools for monitoring the functional groups in the structures of macromolecular polymers. As shown in Figure 3C,D, the functional groups and chemical bonds of the obtained starches were the same due to the similar absorption bands detected in FT-IR and Raman spectra, which indicated that the chemical structure of the starch was not altered by fermentation. However, the ratio of the 1047 cm^−1^/1022 cm^−1^ band (DO) characterizing the changes in the crystalline and amorphous regions of the starch granules [40,41] and the 995 cm^−1^/1022 cm^−1^ band (DD) characterizing the degree of the double helix structure of the starch granules [40] were different between the obtained starches. The back-slopping fermented starch showed the largest values, followed by the starches extracted by lactic acid bacteria fermentation and water extraction, suggesting that the content of the double helix structure and the crystallinity of the starches increased after fermentation, and the effect of back-slopping fermentation was more obvious. Furthermore, the short-range molecular order of the double helix of the starch sample can also be characterized by the full width at half maximum (FWHM) value of the Raman band at 480 cm^−1^ [26], and smaller FWHM values indicated higher relative crystallinity in the starch. As can be seen from Table 3, the order of the FWHM values of the Raman bands at 480 cm^−1^ for the three starches was the water extracted starch, lactic acid bacteria fermented starch, and back-slopping fermented starch (*p* < 0.05), which were in agreement with FT-IR spectra results. These results all suggested that there was a decrease in the amorphous region and an increase in the double helix content of the fermented starches.

### 3.5. Pasting and Thermal Properties

The pasting properties of the starches are shown in Table 4. The viscosity of the starch extracted by fermentation was higher than that of the starch extracted by water. The viscosity of the starch extracted by back-slopping fermentation was higher than that of the starch extracted by lactic acid bacteria fermentation, which may be related to the ratio of B1 chains and particle size distribution. A previous study also suggested that the viscosity of the starch extracted by lactic acid bacteria fermentation increased with an increase in the proportion of B1 chains [42]. Moreover, the relatively smaller particle size could also result in a higher starch viscosity [43]. In addition, the fermented starches had higher breakdown values than the starch extracted by water extraction, which may be related to the higher viscosity and swelling power [43,44,45]. The setback reflects the rearrangement of amylose leached from swollen starch granules during cooling, which is closely related to the properties of starch gels [42]. The value of setback was significantly increased in the fermented starch, especially in the back-slopping fermented starch.

Thermal properties of the gelatinization start temperature (T_0_), peak temperature (T_P_), gelatinization end temperature (T_c_), and gelatinization enthalpy (ΔH) of the obtained starches are shown in Table 4. The T_p_ of the obtained starches was similar and did not show significant difference (*p* > 0.05), while T_0_ of the fermented starches was lower than the non-fermented starch as the moisture was more likely to enter the fermented starch during the gelatinization process [16]. In addition, the severe destruction of the amorphous region and outer layer of the crystallization zone of the starch obtained by back-slopping fermentation may induce its lower pasting and onset temperature. Furthermore, the T_c_ and ΔH of the fermented starch were higher than the non-fermented starch, which was correlated with the relative crystallinity and the double helical structure content of the starches [45,46]. However, the T_c_ and ΔH of the back-slopping fermented starch with high relative crystallinity were lower than that of the starch extracted by lactic acid bacteria fermentation. This may be due to the formation of new structures during the rapid retrogradation of amylose, and higher temperature and energy were required to disrupt these structures during gelation [19].

### 3.6. Starch Solubility (SA) and Swelling Power (SP)

The solubility (SA) and swelling power (SP) of the obtained starches are presented in Table 4. The solubility of the fermented starches was higher than that of the non-fermented starch, which may be related to the high content of amylose in the fermented starches [47]. In addition, the swelling power of the fermented starch was also significantly higher than that of the non-fermented starch, especially the back-slopping fermented starch, and this may be related to the increase in the surface area and the number of pores of starch granules in the fermented starches [17]. The characteristics of the solubility and swelling power of the common vetch starch indicated that it could be used as an excellent starch raw material for the production of vermicelli and jelly, and could also be used as a stabilizer and filler in the food industry.

### 3.7. Correlation Analysis

**Figure 4:**
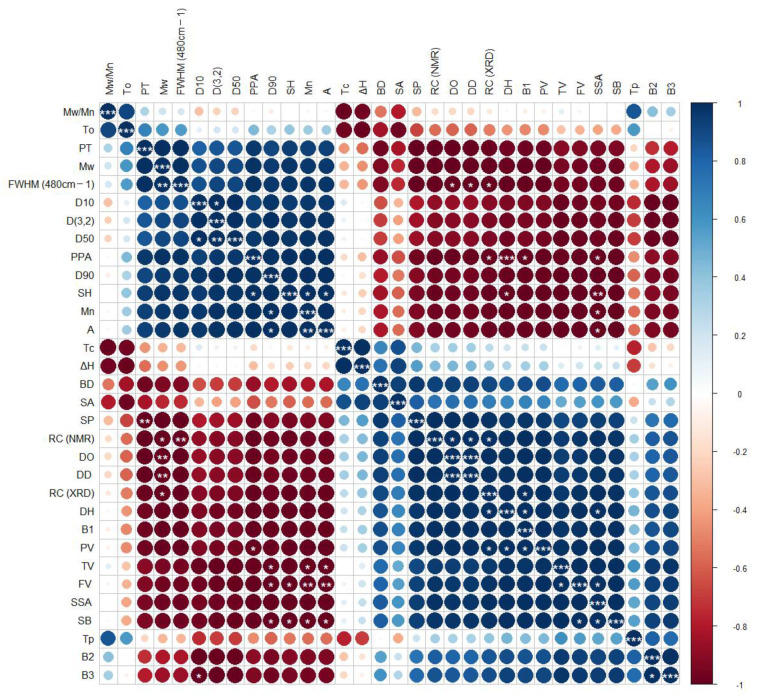
Correlation heat map of different parameters of starch samples extracted by different methods. * is *p* ≤ 0.05, ** is *p* ≤ 0.01, and *** is *p* ≤ 0.001.

Correlation analysis (physicochemical indexes, structural properties, thermal characteristics, and pasting properties) was performed to further investigate the effects of different extraction methods on the properties of common vetch starches. As demonstrated in Figure 4, molecular weight, particle size distribution, PPA, and the proportion of A chain were clustered together, while the distance between crystal structure indicators, thermal properties, pasting properties, swelling power, solubility, the proportion of B chains, and SSA was close. There was a significant negative correlation between pasting properties and molecular weight, A chain, and particle size distribution (*p* < 0.05). Similar results were also found in annatto seed starch and corn starch [48]. It can also be concluded that the relative crystallinity of the starch was positively correlated with the degree of order (DO) and double helix structure content significantly (*p* < 0.05). In addition, there was a significant negative correlation between the molecular weight and pasting viscosity; whereas, the specific surface area and pasting viscosity showed a significant positive correlation (*p* < 0.05), which suggested that the starch granules extracted by the fermentation method were damaged and had higher gelatinization properties. There was a significant negative correlation between the swelling power and pasting temperature (*p* < 0.05), indicating that earlier viscosity onset was associated with an increase in swelling power. These results suggested that the variations in solubility, swelling power, pasting properties, and thermal properties of the obtained starches were induced by changes in the granule morphology, molecular weight, refined structure, and crystalline structure affected by fermentation.

## 4. Conclusions

Water extraction, lactic acid bacteria fermentation, and back-slopping fermentation were all effective methods for extracting common vetch starch, and the purity of the obtained starch was higher than 90%. There was no significant difference in the composition, chemical bonds, and type of crystal structure of the starches extracted by different methods, but fermented starches had higher amylose content, eroded particle surface, smaller molecular weight, larger specific surface area, a higher proportion of amylopectin B1 chain, and lower proportion of A chain. In addition, the fermented starches had higher crystal order and double helical structure content, and higher relative crystallinity. This indicated that fermentation destroyed the surface morphology and changed the internal refined composition and structure of the starch granules, leading to higher solubility, swelling power, enthalpy change, and pasting viscosity of fermentation-extracted starches. Moreover, compared with the starch extracted by lactic acid bacteria fermentation, the starch extracted by back-slopping fermentation had lower amylose content, smaller particle and molecular weight, a larger proportion of B1 chain, larger specific surface area, and formed C_B_-type crystals with higher-order, double helix content, and relative crystallinity, suggesting that the surface erosion was more serious, and the structure dominated by A-type crystals in the outer layer of the crystallization zone was destroyed in the back-slopping fermented starch. These results induced higher swelling power and pasting viscosity and lower enthalpy values. The morphology, internal refined composition, and crystal structure of the starch granules were comprehensively regulated during the extraction of fermentation, especially by back-slopping fermentation. This study provides very useful information for the future use of common vetch starch in the food industry.

## Figures and Tables

**Table 1:** Physicochemical compositions of starch samples extracted by different methods.

Content	WS	LS	NS
Total starch	91.37 ± 0.53 ^a^	92.56 ± 0.39 ^a^	91.98 ± 0.18 ^a^
Moisture	4.85 ± 0.05 ^a^	4.85 ± 0.05 ^a^	4.83 ± 0.18 ^a^
Lipid	0.86 ± 0.05 ^a^	0.77 ± 0.12 ^a^	0.52 ± 0.01 ^b^
Protein	1.07 ± 0.12 ^a^	0.83 ± 0.12 ^a^	0.93 ± 0.01 ^a^
Amylose	30.25 ± 0.65 ^c^	35.69 ± 0.54 ^a^	32.34 ± 0.33 ^b^

Values with different superscripts within a row are significantly different (*p* < 0.05).

**Table 2:** Particle size distribution, molecular weight, and the chain length distribution of starch samples extracted by different methods.

Content	WS	LS	NS
D (3,2)	27.77 ± 0.09 ^a^	26.44 ± 0.02 ^b^	12.95 ± 0.17 ^c^
D10	18.31 ± 0.26 ^a^	18.25 ± 0.02 ^a^	12.74 ± 0.37 ^b^
D50	28.30 ± 0.02 ^a^	27.85 ± 0.02 ^b^	23.58 ± 0.18 ^c^
D90	43.56 ± 0.59 ^a^	42.42 ± 0.04 ^b^	38.60 ± 0.21 ^c^
SSA (m^2^/kg)	224 ± 1 ^c^	297 ± 2 ^b^	463 ± 13 ^a^
Mw (kDa)	52339 ± 977 ^a^	44042 ± 149 ^b^	35573 ± 372 ^c^
Mn(kDa)	13416 ± 350 ^a^	12309 ± 128 ^b^	9294 ± 55 ^c^
Mw/Mn	3.90 ± 0.11 ^a^	3.58 ± 0.03 ^b^	3.83 ± 0.06 ^a^
DP 6–12 (%)	42.04 ± 0. 04 ^a^	37.62 ± 0. 04 ^b^	25.47 ± 0.16 ^c^
DP 13–24 (%)	35.98 ± 0.21 ^c^	40.84 ± 0.05 ^b^	48.21 ± 0.18 ^a^
DP 25–36 (%)	12.35 ± 0.12 ^b^	11.96 ± 0.01 ^b^	15.20 ± 0.18 ^a^
DP > 37 (%)	9.64 ± 0.14 ^b^	9.58 ± 0.01 ^b^	11.01 ± 0.13 ^a^

SSA is the specific surface area. Values with different superscripts within a row are significantly different (*p* < 0.05).

**Table 3:** Short-range molecular order and long-range crystalline structure of starch samples extracted by different methods.

Content	WS	LS	NS
XRD			
Crystal type	C	C	C
RC (XRD, %)	30.22 ± 0.13 ^c^	34.16 ± 0.20 ^b^	39.43 ± 0.41 ^a^
FT-IR			
DO (1047 cm^−1^/1022 cm^−1^)	1.34 ± 0.00 ^c^	1.41 ± 0.01 ^b^	1.48 ± 0.00 ^a^
DD (995 cm^−1^/1022 cm^−1^)	0.80 ± 0.00 ^c^	0.87 ± 0.01 ^b^	0.94 ± 0.00 ^a^
Raman spectrum			
FWHM (480 cm^−1^)	28.50 ± 0.52 ^a^	24.19 ± 0.60 ^b^	19.59 ± 0.13 ^c^
^13^C-NMR			
RC (NMR, %)	47.32 ± 0.9 ^c^	51.29 ± 1.11 ^b^	55.65 ± 1.23 ^a^
DH (%)	58.97 ± 0.86 ^c^	62.88 ± 1.03 ^b^	69.81 ± 0.77 ^a^
PPA (%)	5.91 ± 0.22 ^a^	4.72 ± 0.22 ^b^	2.60 ± 0.12 ^c^
SH (%)	1.93 ± 0.02 ^a^	1.73 ± 0.04 ^b^	1.27 ± 0.11 ^c^

DH is the double helix content detected by ^13^C-NMR, SH is the single helix content detected by ^13^C-NMR, and PPA is the amorphous phase. Values with different superscripts within a row are significantly different (*p* < 0.05).

**Table 4:** Pasting properties, thermal properties, swelling power, and solubility of starch samples extracted by different methods.

Content	WS	NS	LS
PV (cP)	1812 ± 9 ^c^	2055 ± 10 ^a^	1905 ± 17 ^b^
TV (cP)	1518 ± 10 ^c^	1691 ± 21 ^a^	1553 ± 14 ^b^
BD (cP)	295 ± 1 ^b^	364 ± 19 ^a^	352 ± 3 ^a^
FV (cP)	2635 ± 37 ^c^	3126 ± 23 ^a^	2762 ± 25 ^b^
SB (cP)	1118 ± 28 ^c^	1435 ± 2 ^a^	1208 ± 11 ^b^
PT (°C)	77.27 ± 0.29 ^a^	74.47 ± 0.17 ^c^	75.60 ± 0.12 ^b^
To (°C)	63.46 ± 1.16 ^a^	59.93 ± 0.13 ^b^	57.46 ± 0.14 ^c^
Tp (°C)	68.39 ± 0.66 ^a^	68.78 ± 1.16 ^a^	67.59 ± 0.99 ^a^
Tc (°C)	75.24 ± 0.51 ^c^	77.09 ± 0.64 ^b^	80.50 ± 0.19 ^a^
ΔH (J/g)	15.61 ± 0.15 ^c^	17.69 ± 0.10 ^b^	20.17 ± 0.31 ^a^
SA (%)	12.47 ± 0.25 ^b^	13.72 ± 0.16 ^a^	14.03 ± 0.23 ^a^
SP	7.17 ± 0.11 ^c^	8.74 ± 0.01 ^a^	8.11 ± 0.07 ^b^

PV is peak viscosity, TV is trough viscosity, BD is the breakdown (BD = PV − TV), FV is final viscosity, SB is setback (SB = FV − TV), and PT is pasting temperature. T_o_ is onset temperature, T_p_ is peak temperature, T_c_ is conclusion temperature, ΔH is the enthalpy change of gelatinization, SP is swelling power, and SA is solubility. Values with different superscripts within a row are significantly different (*p* < 0.05).

## Data Availability

The data presented in this study are available in this article.
